# The Challenges in Using eHealth Decision Resources for Surrogate Decision-Making in the Intensive Care Unit

**DOI:** 10.2196/47017

**Published:** 2024-04-01

**Authors:** Wan-Na Sun, Chi-Yin Kao

**Affiliations:** 1 Department of Nursing, National Cheng Kung University Hospital College of Medicine, National Cheng Kung University Tainan Taiwan; 2 Department of Nursing, College of Medicine National Cheng Kung University Tainan Taiwan

**Keywords:** decision-making, eHealth, intensive care unit, literacy, surrogate, mobile phone

## Abstract

The mortality rate in intensive care units (ICUs) is notably high, with patients often relying on surrogates for critical medical decisions due to their compromised state. This paper provides a comprehensive overview of eHealth. The challenges of applying eHealth tools, including economic disparities and information inaccuracies are addressed. This study then introduces eHealth literacy and the assessment tools to evaluate users’ capability and literacy levels in using eHealth resources. A clinical scenario involving surrogate decision-making is presented. This simulated case involves a patient with a hemorrhagic stroke who has lost consciousness and requires medical procedures such as tracheostomy. However, due to the medical surrogate’s lack of familiarity with eHealth devices and limited literacy in using eHealth resources, difficulties arise in assisting the patient in making medical decisions. This scenario highlights challenges related to eHealth literacy and solution strategies are proposed. In conclusion, effective ICU decision-making with eHealth tools requires a careful balance between efficiency with inclusivity. Tailoring communication strategies and providing diverse materials are essential for effective eHealth decision resources in the ICU setting. Health professionals should adopt a patient-centered approach to enhance the decision-making experience, particularly for individuals with limited eHealth literacy.

## Introduction

### Decision-Making by Patient Surrogates

The mortality rate of patients in intensive care units (ICUs) exceeds that of those in the general wards. The Society of Critical Care Medicine reported a mortality rate ranging from 10% to 29% in adult ICUs [1]. In Taiwan, the mortality rate in ICUs is 11.6% [2]. The patients in ICUs, grappling with impaired cardiopulmonary functions and severe illness, are in critical condition and are typically physically vulnerable or even in an unconscious state [3]. They depend on the health care team for basic daily activities such as eating and toileting. In addition, given their critical condition, a variety of complex medical treatments such as endotracheal intubation and ventilator use, are often required to maintain their vital signs [4]. Coupled with the administration of multiple medications, patients often find it challenging to express their preferences. If patients have not previously provided an advance directive, they must rely on surrogates for medical decisions. Statistics indicate that 95% of surrogates make at least one major medical decision upon the patient’s ICU admission [4]. Another study highlights that surrogates are involved in up to 71% of medical decisions within the first 48 hours of the patient’s ICU stay [5,6], underscoring the prevalence of surrogates in guiding medical choice decisions for critically ill patients. However, the decision-making process places significant pressure on surrogates, forcing them to reach medical decisions quickly in collaboration with health professionals [5,6]. This pressure can lead to conflicts in decision-making and potentially disrupt the harmony of the medical-patient relationship, all exacerbated by the psychological strain of the possible loss of a loved one and time constraints.

Miller et al [4] noted that 30% of surrogates experienced anxiety and depression, and up to 50% even developed posttraumatic stress disorder. In addition, surrogates possess varying levels of information and knowledge regarding medical treatment, which can influence their capacity to assess and participate in medical decision-making, consequently affecting their ability to comprehend information while communicating with health professionals and making decisions on behalf of the patients [7].

However, with the advancement of health care technology and the catalyzing effect of the pandemic, the use of eHealth tools to assist surrogates in decision-making has become a trend. However, it may pose several challenges. For instance, surrogates may require time to learn to use these eHealth tools effectively [8,9]. Health care professionals must provide effective training and support to ensure that surrogates can fully use these tools. During the use of eHealth tools, there should also be a mechanism for immediate clarification if any doubts arise [8,10]. Furthermore, despite the use of eHealth tools, face-to-face communication remains essential for surrogates. Through in-person observations, one can understand and clarify considerations related to medical interventions stemming from different cultures and family backgrounds [10]. This is vital to genuinely assist surrogates in making informed decisions.

### Use of eHealth Tools in Decision-Making

The term “eHealth” has gained popularity. In 2016, the World Health Organization (WHO) introduced the term to encompass the latest advancements in medical technology, particularly the use of information and communication technology (ICT) to enhance the function and efficiency of the health care system. eHealth encompasses more than just internet medicine; it also includes telehealth and telemedicine. It is a dynamic field that is in a constant state of evolution, signifying not only technological progress but also shifts in how we envision improving the health care landscape as well [11,12].

Scholars regard eHealth as an emerging domain of this generation. It is considered a highly convenient, easily accessible, and user-friendly tool that captures the attention of users [11,12]. Among the most widely used eHealth services is the dissemination of health care information through means such as QR codes, videos, and websites [9]. Many developing countries are transitioning away from paper-based methods, integrating hospital data through ICT, replacing conventional patient care models, and using technology to alleviate the workload on nursing staff to reduce unnecessary costs for individuals and society and alleviate economic and medical burdens. Research has revealed that over 50% of users seek health- or disease-related information through eHealth while up to 60% make decisions based on the information they find [8]. Moreover, in high-income countries, nearly 80% of adults regularly use the internet to access health information [10].

Furthermore, by disseminating pertinent medical and disease-related information through internet platforms and other channels, health professionals can enhance health care knowledge among the public, patients, and their families. This fosters greater awareness of illnesses and facilitates immediate diagnosis and treatment for health concerns, which significantly augments health literacy and diminishes the number of patients facing serious illnesses [10]. In recent years, there has been extensive use of the patient decision aid tools developed using eHealth technology. Some scholars even highlight the potential for eHealth to enhance equitable medical care for remote areas and among patients with limited mobility [10].

Nevertheless, health professionals should carefully consider whether eHealth is suitable for all groups and diseases as a decision-making tool. This is an issue that warrants our attention, as indiscriminate use across all demographics might not be advisable.

### Challenges of Applying eHealth Tools

The application of eHealth tools can present a range of challenges, including economic disparities, information inaccuracies, and the potential exclusion of specific groups. For instance, government agencies should formulate policies and ensure the provision of adequate equipment. Failure to do so may result in unequal access to eHealth tools, favoring higher socioeconomic groups while leaving lower socioeconomic groups without access. This misalignment with the principle of equitable health care is a concern, as some medical institutions may initially refuse to offer services for marginalized groups such as Black communities and low-income individuals [9]. Consequently, a comprehensive support system should be in place before the implementation of eHealth tools.

When conveying medical and disease-related information, it is crucial to consider the risk of patients’ privacy being compromised or the possibility of conveying inaccurate and misleading messages [9]. For instance, Ruppert et al [13] searched for medical information on the social platform YouTube, focusing on terms such as skin cancer and sunscreen. Their findings indicated that up to 40% of the messages contained misinformation and 20% of the messages were potentially misleading [13]. Swartzendruber et al [14] explored the Women’s Resource Center of China and found that up to 53% of the content provided incorrect information about breast cancer treatment, with 84% of the website content being dedicated to advertising programs.

These findings underscore the fact that, despite the eHealth revolution, there are still numerous unresolved issues that require attention. In addition to effectively harnessing technology for caregiving, careful planning is essential to address the potential pitfalls associated with technology. In this era, where eHealth is a prevailing trend, it is crucial to consider how we can make better and more user-friendly use of it. Therefore, there is a need for tools to assess the suitability of patients for using eHealth tools.

### eHealth Literacy and Assessment Tools

As medical technology advances, an increasing number of countries are actively promoting national health literacy through ICT. However, the main purpose of using eHealth technologies is to empower users to make informed health decisions by finding, comprehending, and discerning relevant health information, and then applying this knowledge to address health-related concerns [15]. It is important to note that eHealth differs from traditional health literacy in that it entails using electronic resources to access, understand, and distinguish pertinent health information and then apply this knowledge to deal with health issues [15,16]. It is a dynamic process influenced by factors such as the user's background, knowledge, educational level, and health status, and it can lead to different motivations and strategies in various contexts [15,17].

However, if only a limited number of people participate in or use the eHealth tools, the goal of universalization for these tools is compromised; accordingly, it is important to assess users’ capability and literacy level to provide them with appropriate eHealth tools to enhance their health literacy.

**Figure 1:**
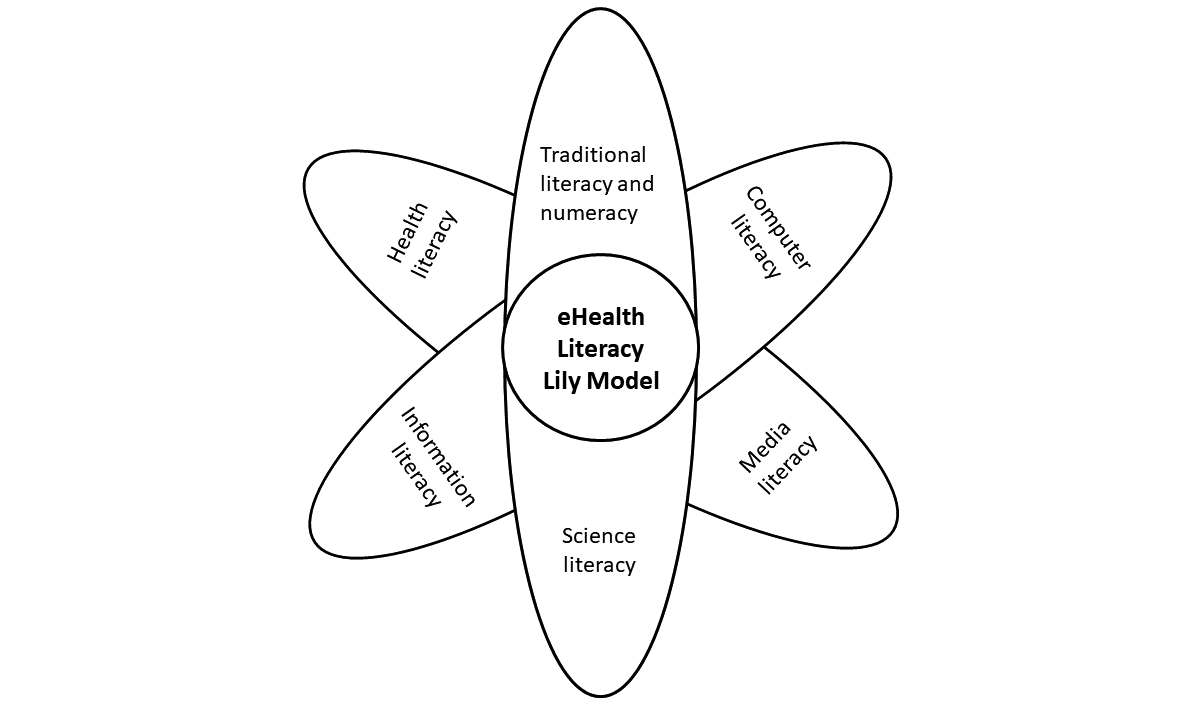
eHealth Literacy Lily Model.

Norman and Skinner [15] introduced the eHealth Literacy Lily Model (Figure 1), which consists of 2 categories, analytic and context-specific. The model comprises 6 various components of literacy: traditional, information, media, health, computer, and scientific. The analytic components (traditional, information, and media) mainly evaluate the users’ fundamental skills and are not directly tied to the content of health literacy. In contrast, the context-specific components (health, computer, and scientific) are more focused on context-specific and computational skills. The following section elaborates on these 6 components of the eHealth Literacy Lily Model. Table 1 provides more details.

**Table 1:** Definitions of the eHealth Literacy Lily Model.

Central types and 6 components		Definition
**Analytic type**		
	Traditional literacy	The most fundamental general abilities include reading and writing skills, and the ability to express oneself logically through reading.
	Information literacy	The ability to find information online or in other ways to address the health literacy issues required by an individual, and even in such a way that others can learn from them.
	Media literacy	The media would consider sociocultural background, market demand, political considerations, and other factors when delivering health literacy messages. Media literacy pertains to whether we can perceive and think critically about such information. If users are unable to understand and organize the content of health information and knowledge from the media, or even develop subjective judgments, they are inadequate in this core skill.
**Context-specific type**		
	Health literacy	The Institute of Medicine in the United States points out that health literacy affects one’s ability to engage in appropriate self-care, which is a necessary skill to interact with the health system, including basic reading and calculating skills.
	Computer literacy	A necessary skill in eHealth, which refers to problem-solving with the use of computer devices and 3C products (computers, communications, and consumer electronics) currently available on the market, for example, desktops, iPads, and mobile phones and the use of software such as email and social network apps.
	Scientific literacy	It pertains to the understanding of natural science as well as the aims, methods, and limitations of scientific research.

Substantiating science-based online health messages in health literacy is a common approach, but it can be a significant challenge for individuals who lack adequate education. This is particularly true since users without these 6 core skills could face difficulties or obstacles when trying to adopt eHealth [9,15]. For this reason, assessing users’ health literacy has become an essential step before implementing eHealth, and the availability of quantitative data enables health professionals to provide evidence-based and useful eHealth tools for users.

Norman and Skinner [15] developed the eHealth Literacy Scale in English, although it has been subsequently translated into multiple languages including Amharic, traditional and simplified Chinese, Dutch, German, Greek, Hebrew, Hungarian, Indonesian, Italian, Korean, Persian, Polish, Portuguese, Norwegian, and Swedish [18]. The questionnaire consists of 8 questions rated on a 5-point Likert scale, ranging from 1 for “Not useful at all” to 5 for “Very Useful.” A total score range is 0-40, with higher scores indicating a better ability to use eHealth. A score of ≤31 points indicates a lower level of eHealth literacy. Previous studies have reported a high internal consistency coefficient α of .88 [19,20]. Further details are provided in Table 2.

**Table 2:** The eHealth Literacy Scale.

No	Items	1^a^	2^b^	3^c^	4^d^	5^e^
1	I know how to find helpful health resources on the internet.	□	□	□	□	□
2	I know how to use the internet to answer my health questions.	□	□	□	□	□
3	I know what health resources are available on the internet.	□	□	□	□	□
4	I know where to find helpful health resources on the internet.	□	□	□	□	□
5	I know how to use the health information I find on the internet to help me.	□	□	□	□	□
6	I have the skills I need to evaluate the health resources I find on the internet.	□	□	□	□	□
7	I can tell high quality from low-quality health resources on the internet.	□	□	□	□	□
8	I feel confident in using information from the internet to make health decisions.	□	□	□	□	□

^a^1: not useful at all.

^b^2: not useful.

^c^3: unsure.

^d^4: useful.

^e^5: very useful.

## Clinical Scenario

### Overview

In this section, we present a simulated clinical scenario to address the challenges associated with applying an eHealth tool. We then use the eHealth Literacy Lily Model and the eHealth Literacy Scale to analyze the situation and propose potential solutions.

Yuliya, a 68-year-old woman, lives with her husband, Eric. Their 2 children are working abroad, and they communicate via internet phone calls. Yuliya does not have any underlying medical conditions and regularly attends government-provided health checkups, following the advice of her neighbors. Her primary sources of health information are her children and neighbors.

Eric, on the other hand, has a complex medical history, including diabetes, chronic kidney disease stage IV, and congestive heart failure stage 2 according to the New York Heart Association. In 2020, Yuliya returned home to find Eric unconscious. After Eric was sent to the emergency room, his cognitive alertness was unclear, EVM of Glasgow Coma Scale (GCS) was E1V2M3; he was then diagnosed with COVID-19 (cycle threshold value 19.5 / polymerase chain reaction showed positive) and acute respiratory failure with chest x-ray showing pneumonia and SpO_2_ range of 77%-82%. The doctor performed tracheal intubation with a ventilator, and Eric was subsequently transferred to the ICU. The doctor informed Yuliya that Eric's consciousness had not recovered and that he needed a computed tomography (CT) scan. The doctor hoped that Yuliya could make an immediate decision regarding the CT scan after the explanation. However, Yuliya was terrified and did not understand the doctor's explanation; nevertheless, she consented to the CT scan. Sadly, Eric was diagnosed with a hemorrhagic stroke of basal ganglia, and even after 2 weeks, he was still unable to breathe spontaneously due to the stroke and he remained in an unconscious state. The doctor recommended a tracheostomy for Eric, proposing that Eric needed long-term care in the future. However, Yuliya was faced with a difficult decision regarding the procedure.

To facilitate Yuliya’s understanding, the nurse provided her with a QR code to access a video. The nurse emphasized that the video contained information about tracheostomy procedures and care. Yuliya was instructed to watch the video and decide whether Eric should undergo the tracheostomy. However, Yuliya encountered challenges as she was unfamiliar with how to use the QR code, and she had limited literacy skills, with her only means of communication being in Taiwanese. Additionally, the video was in Chinese, which further compounded her difficulties. This left her with numerous unanswered questions and uncertainties.

Due to the time difference, Yuliya could not communicate with her children abroad in a timely manner, so upon returning home, Yuliya sought assistance from her neighbors to watch the video, but unfortunately, her neighbors could not operate the QR code either. Instead, they shared their limited knowledge of tracheostomy surgery, drawing from soap opera scenarios. This misconception led Yuliya to believe that patients who had strokes must be bedridden and entirely dependent on others for care.

Meanwhile, the Health Bureau contacted her and requested her to use a social distancing app to monitor Eric’s condition. Yuliya informed the contact person that she did not know how to use mobile phone apps or browse the internet.

### Analysis of the Problem

After evaluating this case using the eHealth Literacy Lily Model and the eHealth Literacy Scale, it became clear that Yulia faced challenges in 6 components of literacy (Table 3). Additionally, Yuliya’s total score on the eHealth Literacy Scale was 8, indicating her limited ability to use eHealth resources effectively.

**Table 3:** Application of the eHealth Literacy Lily Model for the clinical scenario.

Central types and 6 components		Clinical scenario
**Analytic type**		
	Traditional literacy	Yuliya had limited literacy skills, with her only means of communication being in Taiwanese.
	Information literacy	Yuliya often communicated with her children and her neighbors to access health-related information. However, due to the time difference, she could not reach her children promptly. As a result, she discussed tracheostomy surgery with her neighbors, who had limited knowledge, primarily based on soap scenarios.
	Media literacy	Yuliya and her neighbors shared their limited knowledge of tracheostomy surgery, drawing from soap opera scenarios. This misconception led Yuliya to believe that patients who had strokes must be bedridden and entirely dependent on others for care.
**Context-specific type**		
	Health literacy	Yuliya does not have any underlying medical conditions and regularly attends government-provided health checkups, following the advice of her neighbors. Her primary sources of health information are her children and neighbors.
	Computer literacy	Yuliya did not know how to use mobile phone apps or browse the internet.
	Scientific literacy	Yuliya’s medical knowledge was primarily derived from her neighbors, soap operas, and her children, though the accuracy of this information could not be guaranteed.

## Solution Strategies for This Clinical Scenario

### According to the Analytic Type

#### Overview

For individuals with limited traditional, information, and media literacy skills, it is highly recommended to facilitate communication through family meetings. Family meetings can provide comprehensive opportunities for health professionals, patients, or decision makers to exchange information, explain treatments, and clarify questions [21]. Effective communication skills are crucial in this process, and health professionals should use listening skills, questioning skills, and effective message delivery to enhance understanding and facilitate shared decision-making.

#### Listening Skills

Health professionals should encourage patients or surrogates to express their thoughts and concerns. By creating a safe space for open dialogue, they can increase the patients’ or surrogates’ willingness to communicate and provide emotional support. Observing nonverbal cues and summarizing patient or surrogate expectations can help ensure effective communication.

#### Questioning Skills

The use of open-ended and closed-ended questions is essential to discuss and clarify the patient's preferences and concerns. This approach allows health professionals to gain insight into patients’ or surrogates’ thoughts and needs.

#### Sending Messages

Health professionals must provide clear and understandable explanations of treatment options, avoiding the use of medical jargon or technical terms [22]. By offering evidence-based information and using empirical medical research findings to illustrate the advantages and disadvantages of each treatment option, they can empower patients to make informed decisions [23].

In this clinical scenario, the health professionals did not provide an appropriate environment or sufficient time for Yuliya to discuss Eric's medical treatment and address her concerns. Instead, they simply offered a QR code to access a video, assuming it would efficiently convey the necessary information. By using effective communication strategies during family meetings and providing patient-centered explanations and support, health professionals can significantly improve the decision-making process for patients with limited eHealth literacy.

### According to the Context-Specific Type

Individuals with health, computer, and scientific literacy deficits might face significant challenges in accessing essential medical information, potentially leading to difficulties and delays in making informed medical decisions. To enhance individuals’ health literacy in such cases, the government can use various strategies including television advertisements, hospital promotions, public health clinic campaigns, and even celebrity endorsements to increase public awareness and understanding of health-related topics [2].

In the presented clinical scenario, health professionals could take a proactive approach by providing Yuliya with various prepared materials such as notebooks, pictures, DVDs, videos, QR codes, and apps. They should offer guidance on using these tools effectively and assess Yuliya’s understanding of the information provided by eHealth decision resources. The development of decision aids should adhere to specific criteria, including a rigorous and systematic process, the provision of scientific information and data on medical options, assistance in clarifying decision makers’ intentions and preferences, transparency regarding potential conflicts of interest, the avoidance of biased language that might lead to the selection of a particular medical option, and the exclusion of medical jargon or technical terms. Decision aids should provide evidence-based information to empower patients or surrogates to make informed choices [24].

Given the current global trend of an aging population, such decision-making aids are suitable for individuals older than 60 years, as they often have a higher demand for health care information. When creating these aids, it is essential to consider 3 critical components, economic costs, health and recovery aspects, and social and environmental support. Despite the increasing availability of technology-based decision-making aids, printed materials in large print, colorful chart cards, or brochures remain the most widely accepted format among patients and their surrogates, especially for more elderly populations [25].

### Summaries of This Case

In this clinic scenario, health professionals did not consider Yuliya’s eHealth literacy when providing appropriate resources, even though she needed to make immediate decisions. To address this issue in the future, we recommend the following actions: first, health professionals should take the time to explain the conditions for a patient's admission to the ICU thoroughly. This includes providing clear information about the patient’s medical condition, the available treatment options, and the potential risks and benefits associated with each choice. Second, when circumstances permit, health professionals should engage in open and transparent communication with patients and their families. They should offer opportunities for patients to ask questions and express their concerns regarding daily urgent medical treatment decisions. Third, it is crucial to provide Yuliya and patients like her with sufficient and understandable information that empowers them to make informed decisions about their health care. This may involve using plain language, visual aids, or written materials to enhance their understanding.

By implementing these recommendations, health professionals could improve the overall patient experience and facilitate more effective shared decision-making, especially for individuals with limited eHealth literacy like Yuliya.

### Recommendations for eHealth Issues in ICUs

Based on current development trends, the use of eHealth tools has become an irresistible trend. To address this issue in the future, we recommend the following actions: (1) education and training: ensure that all health professionals receive appropriate training on how to use eHealth tools; (2) when developing eHealth tools, it is crucial to ensure that the provided content is accurate, aligns with the needs and expectations of surrogates, and enhances usability through a user-friendly interface. Additionally, having real time technical support for clarification is essential to prevent any disruptions for surrogates during usage; and (3) conduct regular assessments of the eHealth tools and establish an evaluation framework that incorporates data and feedback for continuous updates and adjustments based on the surrogates' needs.

In summary, effectively integrating eHealth tools can improve the quality and efficiency of ICU care. However, it is imperative to ensure that these tools not only meet medical standards but also consider patient privacy and data security. Therefore, strategies such as education, interoperability, security, and user-friendliness are key to successfully meeting surrogates' expectations with eHealth tools.

## Conclusions

In conclusion, the challenges associated with using eHealth decision resources to assist surrogates in ICU decision-making are multifaceted and require careful consideration. The high mortality rate in ICUs, when coupled with critical conditions, compels swift decision-making by surrogates, and the increasing popularity of eHealth tools adds complexity to this process.

The application of eHealth tools brings both opportunities and challenges. While these tools have the potential to disseminate important medical information and improve health care knowledge, they also present economic, informational, and inclusivity challenges. Economic disparities, information inaccuracies, and the risk of excluding specific groups must be addressed to ensure equitable access and benefit for all.

The eHealth Literacy Lily Model and the eHealth Literacy Scale are valuable tools for assessing individuals’ capability and literacy levels in using eHealth resources. To improve decision-making for individuals with limited eHealth literacy, both analytic and context-specific approaches are recommended. Effective communication through family meetings via using listening and questioning skills and providing patient-centered explanations can all enhance understanding and facilitate shared decision-making. Additionally, proactive measures such as using various materials, realia, and decision-making aids, can improve literacy in the health, computer, and scientific dimensions.

In summary, recognizing the time constraints and high-stakes nature of ICU decision-making, it is crucial to strike a balance between leveraging eHealth tools for efficiency and ensuring inclusivity. Health professionals should adopt a patient-centered approach, considering the diverse literacy levels and needs of individuals, especially those with limited eHealth literacy. Tailoring communication strategies, providing diverse materials, and assessing eHealth literacy levels using tools like the eHealth Literacy Scale are essential steps toward enhancing the effectiveness of eHealth decision resources in the ICU setting.

## Abbreviations

CT: computed tomography
GCS: Glasgow Coma Scale
ICT: information and communication technology
ICU: intensive care unit
WHO: World Health Organization
